# Expensive care? Resource-based thresholds for potentially inappropriate treatment in intensive care

**DOI:** 10.1007/s40592-017-0075-5

**Published:** 2018-01-18

**Authors:** Dominic Wilkinson, Stavros Petrou, Julian Savulescu

**Affiliations:** 1 Oxford Uehiro Centre for Practical Ethics, Faculty of Philosophy, University of Oxford, Oxford, UK; 2 John Radcliffe Hospital, Oxford, UK; 3 Warwick Medical School, University of Warwick, Coventry, UK

**Keywords:** Medical ethics, Intensive care, Medical futility, Withholding treatment, Cost-benefit analysis

## Abstract

In intensive care, disputes sometimes arise when patients or surrogates strongly desire treatment, yet health professionals regard it as potentially inappropriate. While professional guidelines confirm that physicians are not always obliged to provide requested treatment, determining when treatment would be inappropriate is extremely challenging. One potential reason for refusing to provide a desired and potentially beneficial treatment is because (within the setting of limited resources) this would harm other patients. Elsewhere in public health systems, cost effectiveness analysis is sometimes used to decide between different priorities for funding. In this paper, we explore whether cost-effectiveness could be used to determine the appropriateness of providing intensive care. We explore a set of treatment thresholds: the probability threshold (a minimum probability of survival for providing treatment), the cost threshold (a maximum cost of treatment), the duration threshold (the maximum duration of intensive care), and the quality threshold (a minimum quality of life). One common objection to cost-effectiveness analysis is that it might lead to rationing of life-saving treatment. The analysis in this paper might be used to inform debate about the implications of applying cost-effectiveness thresholds to clinical decisions around potentially inappropriate treatment.

## Introduction

There are many ethical dilemmas in intensive care. However, one of the most challenging questions is the following: when are professionals justified in refusing a family’s (or patient’s) request to provide life-prolonging treatment such as intubation, mechanical ventilation and multi-organ support? For example, a critically ill patient may have an extremely low chance of survival if intensive care is provided, or treatment may be associated with a prohibitively high cost, or survival may be possible only in a state of substantially reduced quality of life. In such situations, physicians may believe that intensive care should not be provided. But how low a chance of survival would justify such a determination? How costly is too costly? How low a quality of life is too low?

There are arguably two key ethical justifications for physicians declining requests for treatment. It may be justified to refuse to provide treatment either where that treatment would harm the patient, (that is, it is against the interests of the patient), or where the treatment would harm others through the unfair consumption of scarce resources in a public health system (Wilkinson and Savulescu 2011). In this paper, we set aside the former to focus exclusively on desired treatments of potential benefit, i.e. those that are arguably in a patient’s best interests. We explore the question of harm to others, drawing on an assessment of cost-effectiveness, often used elsewhere in medicine, to define the limits of appropriate treatment in intensive care.^1^ The aim of this analysis is to shed light on the role of resource limits in treatment decisions, to explore what the implications would be for intensive care and to identify key normative and empirical questions that need to be addressed before constructing policy or guidance in this area. We focus on the specific clinical setting of intensive care—since that allows focused examples of how cost-effectiveness might be applied. The intensive care unit is also a frequent location for disputes about potentially inappropriate treatment. We will start by providing background on futility/potentially inappropriate treatment and cost-effectiveness in medicine (Sects. 2 and 3). In Sects. 4–6 we will apply cost-effectiveness to derive thresholds for potentially inappropriate treatment. We will consider concerns and objections to such thresholds in Sect. 7.

## Potentially inappropriate treatment

The concept of medical futility first appeared in the medical literature in the early 1990 s, as a way of resolving disputes about end of life care (Schneiderman et al. 1990). It has a much older pedigree, however: the Hippocratic corpus originally included a promise to avoid medical treatment in patients “overmastered by their disease” (Whitmer et al. 2009).

There is strong professional support for the idea that doctors are justified in not providing treatment if they judge that it would be *futile* to do so. For example, the UK General Medical Council (GMC) guidance on “Treatment and Care towards the end of life” notes: “there is no obligation to give treatment that is futile” (General Medical Council 2010, p. 80).

This raises a further question, though: when is treatment futile, what would justify making such a determination? The GMC guidance does not provide any assistance here; nor, indeed, do many other guidelines. One influential analysis defined treatment as quantitatively futile if treatment had a less than 1% chance of success, or qualitatively futile if it would “merely preserve unconsciousness or fail to relieve total dependence on intensive medical care” (Schneiderman et al. 1990; Schneiderman and Jecker 2011). However, both such definitions have been subject to criticism that the thresholds chosen are arbitrary and value-laden (Truog et al. 1992).

The difficulty in defining futility has led many ethicists to reject the concept, and dismiss its use in treatment decisions (Truog et al. 1992; Brody and Halevy 1995; Helft et al. 2000). Helft, and colleagues, describing the “rise and fall of the futility movement” blame a failure to reach “consensus on a specific definition of futility or on an empirical basis for deciding that further care would be futile” (Helft et al. 2000, p. 295).

There are numerous synonyms for medical futility, including “non-beneficial”, “not clinically indicated” and “medically inadvisable” (Wilkinson 2018). Recent guidance from an international group of 5 critical care societies (‘2015 Consensus statement’) refers to treatment that is “potentially inappropriate” (Bosslet et al. 2015). Unifying these different terms is the following:

*Potentially inappropriate: treatments that have at least some chance of accomplishing the effect sought by the patient, but clinicians believe that competing ethical considerations justify not providing them* (Bosslet et al. 2015).

As already noted, quantitative and qualitative thresholds for futile or inappropriate treatment have been criticized for being arbitrary. For example, what probability of successful treatment is so low that it becomes inappropriate—10%, 1%, or 0.1%? Although medical professionals may regard treatment as not worth providing if it has a < 1% chance of ‘success’, patients may have different views. If the alternative is death, some may prefer treatment even if there is only a one in a million chance of their life being prolonged. Furthermore, even if we all agreed on a particular statistical threshold (say 1%), applying this in a uniform way to treatments appears to ignore relevant differences between cases. For example, it would arguably be reasonable to provide a desired simple, inexpensive, innocuous treatment that might save a patient’s life, but is very unlikely to do so (for example, if there is only a one in 100,000 chance of this occurring). Conversely, it may be unethical to provide an extremely expensive, high risk and burdensome treatment that has a 15% chance of saving the patient’s life. In that case the 1% threshold seems too low—perhaps such treatment should only be an option if there is a > 50% chance of success?

Such discussions illustrate the importance of determining *why* treatment is judged inappropriate. We have argued previously that there are two core ethical justifications for medical professionals refusing to provide treatment that is desired by the patient (Wilkinson and Savulescu 2011). It would be ethical to decline treatment if either the treatment would be harmful to the patient, *or* if it would be harmful to others. The first of these grounds is particularly important where decisions are being made for non-competent patients, for example for children or newborn infants. Parents’ request for treatment should be overruled if their decision would cause a significant risk of serious harm (Diekema 2004; Wilkinson and Nair 2016). However, even if their decision does not cross the harm threshold, indeed even if it would be overall beneficial, there is a further important question about the cost of treatment (Wilkinson and Nair 2016).

There are different ways that providing medical treatment to one patient might harm other individuals. It is possible that treating a patient in a particular way, or in a particular place might cause health risks for others.^2^ Alternatively, providing treatment might cause psychological distress to others.^3^ Arguably though, the most likely and most important way that providing treatment to one patient could harm others is through consuming limited resources in a public health system. In intensive care, there are a finite number of physical spaces and staff to care for patients. More broadly, public health systems have finite budgets within which to provide a range of competing medical priorities. All such systems need to make decisions about where their resources will be directed: which illnesses will be treated, which treatments will be provided, and which populations will receive those treatments? Where one patient receives highly expensive treatment, the necessary corollary in a closed public health system is that somewhere else in the system something that is desired cannot be provided.

## Cost-effectiveness

One widely used way of deciding between different priorities for funding in a publicly funded healthcare system is to compare their cost effectiveness. In a cost-effectiveness analysis, the costs of an intervention are divided by its benefits, setting up a cost-effectiveness ratio. Interventions with a lower cost-effectiveness ratio are preferred. Cost-effectiveness analysis takes into consideration two factors that are important to decision-makers. The cost of treatment has direct implications for the number of individuals who are able to benefit from health systems with a fixed budget. When alternative treatments are equally effective (i.e. the denominator in cost-effectiveness comparison is the same), choosing a less expensive treatment simply means that more patients are able to be treated. Where we are contemplating life-saving treatment, cheaper treatment means that more lives will be saved. The *effect* of treatment has implications for the amount of value (usually understood in terms of health benefit) that the health system is able to promote or improve. If the numerator (cost) is the same, cost-effectiveness analysis will favour more effective treatments.

Although the underlying principles are simple, and widely accepted in many countries, translating them into practice is much more complicated (Bognar and Hirose 2014; Wilkinson and Savulescu forthcoming). Assessing the effectiveness of treatments includes factoring in uncertainty and a wide range of possible outcomes. Determining the costs of treatment can include complicated estimation and projection as well as decisions about which costs to include. Comparing different treatments and illnesses, particularly where there is an attempt to quantify effectiveness raises questions about commensurability. Evaluating the impact of treatment invokes difficult and controversial questions about how we should evaluate wellbeing and states of health and disability.

One widely used metric for quantifying the effectiveness of treatment uses the concept of quality-adjusted life years (QALY). The QALY is a preference-based measure of health outcome that combines length of life and health-related quality of life in a single metric. There are a number of vehicles for assessing the cost-effectiveness of treatments, for example, within the context of randomized controlled trials where pertinent data are collected on an individual patient basis, or using decision-analytic models where typically data from multiple sources are synthesized using mathematical techniques. Modelling based economic evaluations usually assign probabilities to branches emanating from chance nodes with endpoints of each pathway given values or payoffs, such as costs, life years or QALYs. These alternative vehicles allow analysts to express the cost-effectiveness of new treatments in terms of incremental cost per QALY gained. Cost-effectiveness assessment using this metric can help to assess which, of two or more different treatments, should be funded. As already noted, other things being equal we have strong reason to choose treatments that are less costly and more effective. Incremental cost per QALY calculations can also be used to decide whether the incremental benefit of an individual treatment is sufficiently great, relative to the incremental cost, to provide it. For this purpose, some countries and policy makers have used cost-effectiveness thresholds to efficiently and consistently decide between different priorities. In the UK, for example, interventions that cost less than a threshold level of £20–30,000 per QALY are usually funded by the National Health Service, while those that cost more than £30,000 are not usually provided (Simoens 2009; Cleemput et al. 2011).^4^

While cost-effectiveness is not routinely used in the United States for health care funding decisions, treatments costing more than USD$100,000–$150,000 are often regarded as not offering reasonable value (Neumann et al. 2014).^5^ Cost-effectiveness has been used to inform some policy decisions around provision of treatment in the US, for example in Oregon (Dubois 2016). It has also been used to justify recommendations in national evidence-based clinical guidelines (Dubois 2016) and by some managed care funds (Sullivan et al. 2015).

There is considerable ethical debate about the use of cost-effectiveness thresholds, and QALYs for deciding between different treatments (Harris 1987; Singer et al. 1995; Nord et al. 2009; McMillan and Hope 2010; Bognar and Hirose 2014). It is not the aim of this paper to review those arguments, to defend cost-effectiveness analysis, nor to assess whether the incremental cost per QALY metric *should* be used to decide between medical treatments. Rather, the point is that cost-effectiveness thresholds are already widely used in many public health systems to decide between different treatments (for example, whether new drugs will be funded). If that approach is justified, on the grounds of consistency, it appears that these same thresholds should be applied to other medical decisions where prioritization is necessary.^6^ What would be the implications of such an approach for decision-making in intensive care? One common objection to cost-effectiveness analysis is that it might lead to denial of potentially life-saving treatment from dying patients. The results of the analysis below might be used to inform debate about what the actual implications would be of applying cost-effectiveness thresholds to clinical decisions around potentially inappropriate treatment.

## Low probability treatments

We started this paper by asking how low a chance of survival is too low? Could cost-effectiveness be used to help answer that question?

The general formula for assessing cost-effectiveness is given by the following:

$${\text{Incremental Cost Effectiveness}} = \frac{{\bar{C}_{2} - \bar{C}_{1} }}{{\bar{E}_{2} - \bar{E}_{1} }} = \frac{\Delta C}{\Delta E}$$

where $\bar{C}_{2}$ and $\bar{E}_{2}$ refer to the mean cost and mean effectiveness of a comparison intervention, and $\bar{C}_{1}$ and $\bar{E}_{1}$ refer to the mean cost and mean effectiveness of the reference intervention. We are interested in comparing the alternatives of continuing intensive care versus withdrawal of intensive care. If we assume that all patients who have treatment withdrawn die, and that there are no costs (nor indeed health consequences) associated with that option,^7^ the formula can be simplified:

$${\text{Cost Effectiveness}} = \frac{{\bar{C}_{2} }}{{\bar{E}_{2} }}$$

The effectiveness of continuing intensive care will depend upon the mean probability of survival ($\bar{p}$), duration of survival (if the patient survives, $\bar{d}_{s}$) and his/her health-related quality of life (hereafter ‘quality of life’ for brevity) ($\bar{q}$).^8^

1 $$\frac{\text{Cost}}{\text{Effectiveness}} = \frac{{{\text{Cost}} (\bar{C})}}{{{\text{Probability of survival}}\,(\bar{p}) \; \times ({\text{Duration of survival }}\left( {\bar{d}_{s} } \right)\; \times \; {\text{Quality of life }}(\bar{q}))}}$$

These variables are not necessarily independent. For example, there is potentially a complex relationship between cost of providing treatment and duration of survival. Patients who survive for a long period of time may incur extra costs as well as gaining extra benefits from treatment (Paulden and Culyer 2010). We will here assume that all of the health care costs in question are up front, and ignore long-term costs.^9^ We will return in Sect. 6.1 to long-term costs and how these might influence decisions.

### Probability threshold

If there is a fixed cost effectiveness threshold (CET), this formula can be transformed to estimate the probability threshold (P_T_) where P_T_ is the lowest probability of survival for appropriate cost-effective life-saving treatment,

2 $$P_{T} = \frac{{\bar{C}}}{{CET \; \times \;\bar{d}_{s} \; \times \; \bar{q}}}$$

We could make some assumptions about each of these values to calculate P_T_ for a hypothetical patient needing intensive care (and who will die without that treatment). To start with, we could assume the following^10^:

CET = £30,000

$$\bar{d}_{s}$$  = 10 years^11^

$$\bar{q}$$  = 1^12^

Based on a 20-day intensive care stay at a daily cost ($\bar{C}_{d}$) £1300/day^13^

$$\bar{C}$$ = £26,000

3 $$P_{T} = \frac{26{,}000}{30{,}000 \times 10} = 0.09\,\,\text{ie}\;9{\text{\% }}$$

In other words, it would be cost-effective to provide 20 days of life-saving intensive care to this hypothetical adult patient as long as they had a chance of survival greater than 9%.

We have used UK currency in this example because of the widespread use of cost-effectiveness analysis in UK health care decision-making. For a hypothetical comparison, we could also apply the US$100,000 threshold. The average daily cost of ICU from a large database of US hospitals in 2002 was $3000/day (for medical intensive care) (Dasta et al. 2005). The corresponding probability threshold would be 6%.

One thing to note about this calculation of the probability threshold is that P_T_ is inversely proportional to the duration of survival. This has implications for different areas of intensive care. Since survivors from neonatal intensive care are likely to live considerably longer than those from adult intensive care, we would expect, other things being equal, P_T_ in the newborn intensive care unit (NICU) to be significantly lower. For example, assuming a full lifespan (70 years) P_T_NICU would be 1%.

## High cost treatment

Even if treatment had a 100% chance of success, it may not be affordable, and consequently might be judged to be unreasonable to provide.

We could calculate the cost threshold (C_T_) for intensive care, where C_T_ is the highest additional cost for appropriate cost-effective life-saving treatment.

4 $$C_{T} = CET\; \times \;((\overline{{{\text{duration}}_{2} }} \; \times \; \, \overline{\text{quality}}_{2} ) - (\overline{{{\text{duration}}_{1} }} \times \, \overline{\text{quality}}_{1} ))$$

In adult intensive care, for a 10 year life expectancy and full quality of life^14^ $C_{T} \left( {AICU} \right) = 30{,}000\; \times \; 10 = 300{,}000$

The corresponding figure based on the US threshold would be US$1,000,000.

### Duration threshold

One practical question faced by clinicians in intensive care is how long it is reasonable to continue treatment. For example, if a patient is still ventilator dependent after 1 month of intensive care, is it time to stop treatment? What about 3 months, 1 year?

If the patient is guaranteed to survive for a full life-span with full quality of life, we can work out the maximum duration of treatment, the duration threshold.

5 $${D_{T}} \left( {AICU} \right) = \frac{{CET \; \times \;\bar{d}_{s} \; \times \; \bar{q}}}{{ {\bar{C}_{d}} }} = \frac{30{,}000\; \times \;10\; \times \;1}{1300} = 231 \;{\text{days}}$$

By comparison, (unsurprisingly) the duration threshold for newborn intensive care, would be much longer at 1615 days.^15^

However, this appears overly simplistic. As well as leading to increasing costs, prolonged duration of treatment would appear to reduce the chance of survival. Table 1 combines the probability of survival, and duration of survival to derive D_T_.

**Table 1:** Duration threshold (in days) for maximum cost-effective intensive care based on different estimates of probability of survival (p) and duration of survival

P	NICU	PICU	AICU
0.1	162	138	23
0.25	404	346	58
0.5	808	692	115
0.75	1212	1038	173

*NICU* neonatal intensive care unit, predicted survival 70 years, *PICU* paediatric intensive care unit, predicted survival 60 years, *AICU* adult intensive care unit, predicted survival 10 years

It is difficult to estimate the chance of survival for patients who have prolonged intensive care stay. It might be expected that survival rates would be low. However, survival figures may well be influenced by self-fulfilling prophecies.^16^ If the probability of survival with continued treatment is 50%^17^ the Duration threshold is over 2 years of mechanical ventilation for a newborn infant and more than 3 months for an adult depending on the predicted duration of survival.^18^

## Reduced quality of life

The above analysis ignores the substantial anticipated reduction in quality of life for patients sick enough to require prolonged intensive care. We have hitherto set aside questions of quality of life, and assumed full quality of life in survivors.

Could cost-effectiveness be used to determine a quality threshold—a level of future or current quality of life sufficiently low that treatment should not be provided?

In cost-effectiveness calculations, ‘q’ represents a way to compare different health states. This evaluation is often denoted ‘health utility’, though it does not reflect usefulness of the health state (or individual), nor does it necessarily reflect a commitment to utilitarianism (Torrance 1987). Health utility reflects population (or individual) preferences for different health states. There are different ways to arrive at values for ‘q’, for example using rating scales, standard gambles or time trade-off. The latter method asks members of the public to trade off length of survival against a health state (often described in terms of a set of attributes, e.g. physical, emotional, sensory, self-care and cognitive function as well as pain) (Torrance 1987; Brazier 2007). As an example, people might be asked if they were prepared to choose a treatment that would lead to survival of 6 months in a state of full health, compared with survival of 12 months in a state of illness or impairment. Choosing this treatment (and therefore foregoing 6 months of survival) would imply evaluating this state as half as good as full health. As an example of values, one study of cost-effectiveness of neonatal intensive care, assumed a value of 0.8 for states of mild, 0.6 for moderate, and 0.4 for severe disability (Doyle 2004). There are controversial questions about how best to derive utility values for health states and whether to apply them in decisions about treatment affordability. There are important questions about whether it is meaningful to discount the value of prolonging life in the setting of reduced quality of life. We will set those aside for now (and return to some of them in Sect. 7). Here, the aim is to explore how these variables would affect treatment thresholds, if used.

### The quality threshold

How would quality of life considerations affect the cost-effectiveness of providing prolonged intensive care? Based on the assumptions outlined above, Table 2 presents incremental cost-effectiveness estimates for a 3-month stay in intensive care at different utility values for subsequent health state.^19^

**Table 2:** Cost-effectiveness of prolonged intensive care at different levels of quality of life (assuming 10 years of survival after intensive care) using UK or US based estimates of the cost of intensive care

Q	Cost/QALY (UK £)	Cost/QALY (USD $)
1	12,090	27,900
0.9	13,433	31,000
0.8	15,113	34,875
0.7	17,271	39,857
0.6	20,150	46,500
0.5	24,180	55,800
0.4	30,225	69,750
0.3	*40,300*	93,000
0.2	*60,450*	*139,500*
0.1	*120,900*	*279,000*

*Q* predicted quality of life

Italic values indicate treatment that exceeds the CET (£30,000 or $100,000)

As seen above, the incremental cost-effectiveness ratio rises above standard thresholds if health utility is rated below 0.3 (below 0.4 in the UK).

We have assumed thus far that patients have some chance of recovery and no longer requiring intensive medical care. However, some patients (for example with spinal cord injury, or progressive neurodegenerative diseases) may require respiratory support or other intensive life-prolonging therapies permanently.

If quality of life is appropriate to take into consideration we could calculate the maximum yearly incremental cost of treatment (C_T_) that would fall within standard cost-effectiveness thresholds.

6 $$ {\text{Cost Threshold}}\; (C_{T} ) = CET \; \times \;\bar{q} $$

For patients with very low quality of life, even relatively inexpensive treatments may exceed the Cost Threshold. At a value for q of 0.1, C_T_ would be £3000.

As an example, in an analysis of health care costs for ex-preterm infants the average cost per year for children with severe disability (once they reached primary school) was approximately £12,000 (Mangham et al. 2009).

If this cost were life long, we could calculate the quality threshold—the lowest value for future quality of life consistent with cost-effective treatment. Such a value could be used in two distinct ways. It could be used to assess a level of predicted disability sufficiently severe that treatment should not be provided. Alternatively, if treatment were provided for individuals with a particular condition, it can be used to infer the value (or the lowest value) attributable to that health state. Using the above figures

7 $$ {\text{Quality Threshold }}\left( {Q_{T} } \right)\;{\text{for ongoing treatment }} = \frac{{\bar{C}}}{CET} = \frac{12,000}{30,000} = 0.4 $$

**Fig. 1:**
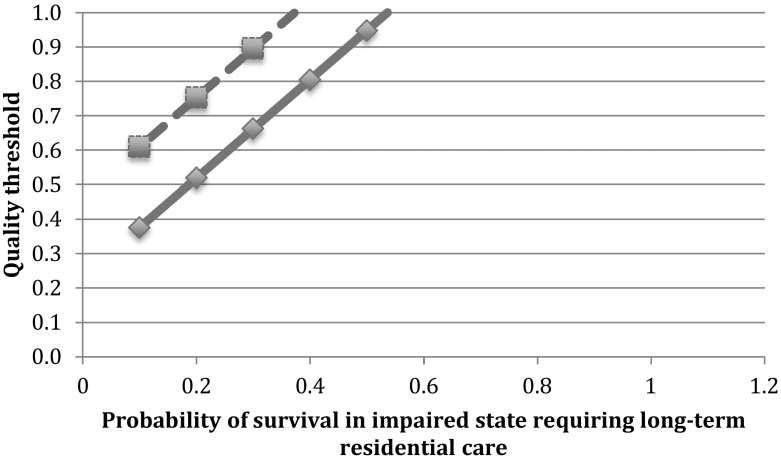
The quality threshold for adult patients requiring possible long-term residential care after an intensive care admission. Solid line—quality threshold for a 1 week intensive care stay (total cost £7000). Dashed line—quality threshold for a two-week intensive care stay. Assuming cost of residential care is £42,853/year. Assuming full probability of survival during and after intensive care stay

The value for Q_T_ would fall if long-term residential care is required. For example, from one estimate, the yearly cost of residential care for severely affected young adults with acquired brain injury was £42,853 per year, (Curtis 2014). Since this value exceeds the CET, it may rule out any life-saving treatment in the setting of certain long-term dependence on residential care. (That might, for example, appear to justify excluding nursing home residents from intensive care admission). However, we could combine probability and quality to determine the quality threshold in the setting of uncertainty (Fig. 1). For example, from the figure, if there is a greater than 50% chance of needing long-term residential care, a short period of intensive care treatment would exceed the UK cost-effectiveness threshold even if quality of life were assigned full value. Conversely, if quality of life (for a patient predicted to require long-term residential care) were assessed to be less than a value of q = 0.5, a short period (1 week) of intensive care would be cost-effective only if the chance of needing residential care were less than 20%.

In the face of uncertainty, one possible cost-effective strategy might be to commence intensive care treatment, then to later withdraw treatment if the chance of poor quality of life and long-term dependence of care were sufficiently high. However, this would only provide a solution if physicians were prepared to withdraw treatment based on resource considerations. There is some evidence that clinicians find this much more challenging than withholding treatment (on the same grounds) (Wilkinson and Savulescu 2012). Furthermore, there is the risk that by the time that outcome is sufficiently certain, the patient is no longer dependent on life-prolonging treatment, and survival (with dependence on care) is likely (Wilkinson 2011).

This analysis highlights three key normative questions that would need to be addressed if a quality threshold were to be used to define a level of disability sufficiently severe that intensive care, or other intensive life-sustaining treatments should not be provided.

First, is it appropriate to exclude patients from life-saving treatment on the basis of disability? Many would regard such a determination as discriminatory (Bognar 2010; Wilkinson and Savulescu forthcoming). However, whether this is justified might depend on the type and level of disability falling outside the quality threshold for treatment (Wilkinson and Savulescu forthcoming).

Second, what health utility should be attributed to life for those who have been born in states of disability, or who have adapted to an acquired disability? Should a higher utility state be used for them because this is their default state? Health economists argue that it is the preferences of the general public who take on the role of citizens that should be used to inform social decision-making (Dolan et al. 2003). But some ethicists have argued that the preferences of disabled individuals for health states should be used in the place of the views of the non-disabled (Nord et al. 2009; Sinclair 2012; Menzel 2014; Dolan et al. 2003)?

Third, should health care services such as the need for long-term residential care be included in our assessment of long-term costs and rationed, or should it only be particular expensive medications/procedures/devices? Conventionally, cost-effectiveness analysis is used to decide about funding for pharmaceuticals or novel medical interventions. The above analysis has factored in the acute medical costs of intensive care. But one implication of providing treatment for individuals with life-long illness or impairment is a corresponding long-term need for support. Should the costs of additional social and medical care be included in evaluation of the cost-effectiveness of life-saving treatment and in thresholds for treatment? Some may regard these as a minimum level of health care provision that should be available to all, regardless of disability (perhaps on the basis of the social value accorded to this care). In that case, perhaps they should be excluded from cost-effectiveness analysis? However, such a determination will have implications for the total costs of a public (or private) healthcare system. Where there is a fixed budget, this will also affect the quality of long-term care available, and the availability of funding for other priorities.^20^

## Should cost-effectiveness thresholds be applied to intensive care?

The above analysis has explored the application of standard cost-effectiveness criteria to decisions about life-sustaining treatment in intensive care. It has highlighted what the implications would be for treatment if such thresholds were applied.

But *should* cost-effectiveness thresholds (CET) apply to potentially life-saving treatments for gravely ill patients?

### The rule of rescue

One reason *not* to use CET in intensive care is because of the ‘rule of rescue’. The idea behind the rule of rescue is that health professionals have a special ethical obligation to provide life-saving treatment (rescue) to specific identifiable individuals that they are caring for. The rule is sometimes thought to justify exceeding population derived rules or guidelines (for example on the basis of limited resources), and providing treatment even if it would normally be regarded as too expensive to do so, or if it resources could be more effectively used for another (anonymous) patient. Plausibly, the rule of rescue provides an explanation for therapies that are sometimes available in intensive care that clearly exceed conventional cost-effectiveness thresholds (Jonsen 1986). The rule of rescue appears to be endorsed by a significant proportion of US intensive care physicians and nurses (Kohn et al. 2011).

The rule of rescue has been criticized on a number of grounds (Cookson et al. 2008; Brock 2015; Garrett 2015; Jecker 2013). Application of the rule of rescue appears inevitably to mean that overall fewer patients will receive beneficial treatment or will have their life saved, because it sanctions providing treatment that is more expensive or less effective than the standard threshold. The rule risks paradoxical decision-making. For example, it appears to condone not funding chemotherapy for cancer if that would exceed the CET, but then permits providing more expensive life-saving treatment like intensive care. We may have a situation of some patients being denied treatment on the basis of cost, deteriorating and becoming more unwell as a result, and then receiving more expensive treatment when they require intensive care (Schöne-Seifert 2009). It is not clear how chemotherapy is relevantly different from intensive care. Both are (in some circumstances) able to save life, or extend life. Both treatments potentially incur substantial cost (for the patient or for the healthcare system), and are either provided or declined to specific identifiable patients.

The rule of rescue appears to be vulnerable to special pleading. Non-identifiable patients denied treatment on the grounds of standard CET might petition their physicians and the media, thereby becoming identifiable individuals who are then able to appeal to the rule of rescue. The National Institute for Health and Care Excellence (NICE) document on social values explicitly rejects application of the Rule of Rescue since it argues that it has a responsibility to anonymous future and present patients (and infers that this responsibility is equal to current non-anonymous patients) (National Institute for Health and Clinical Excellence 2008).

However, even if the rule of rescue is able to resist these criticisms, and were felt to justify giving more resources to intensive care than to other areas of medicine, it is unlikely to sanction providing unlimited resources. It would be highly implausible that intensive care would be granted immunity from consideration of finite public health resources, while all other areas of medicine needed to tighten their belts. More realistically, if we were to give some weight to the Rule, this would simply involve application of a higher threshold for evaluating treatment. In the UK, NICE has recently applied an End-of-Life Premium to give special weight to health gains from certain life-extending end-of-life treatments, for example expensive novel chemotherapy drugs.(McCabe et al. 2015) Effectively, such a premium raises the cost-effectiveness bar for treatments judged to fit into this category. The ethical justification for an End-of-life premium has been criticized, (Cookson 2013) however, if a health system had decided to apply such a premium, it might be used for intensive care, as well as new cancer drugs. In that case, the probability/cost/duration/quality thresholds could still be evaluated, albeit using a different value for the cost-effectiveness threshold.

### Cost-effectiveness and patient characteristics

Another consideration related to the application of CET thresholds to intensive care is the distinction between cost-effectiveness for patient groups, vs cost-effectiveness for individual patients based on their risk, or prognostic profile. Traditionally, cost-effectiveness thresholds have been used to inform decisions about funding, within a public health system, of novel pharmaceuticals or of particular therapies or interventions. However, such thresholds have not by and large distinguished between patient groups (for example, patients with certain characteristics, or with a particular illness) (Dowie 1996; Bognar 2010). If there is a difference between these, such that CET is not appropriately applied to the latter, that may exclude the use of CET for determining treatment thresholds in intensive care.

There might be two different reasons for the distinction between treatments and patients. The first is pragmatic—public health systems need to decide whether novel agents are going to be made available, and need some mechanism for deciding between them. There is a clear and pressing requirement to decide between treatments, in a way that might not apply to groups of patients. The second reason is ethical, a decision to choose one group of patients over another might seem to conflict with principles of equal treatment. It is highly likely that a decision not to provide intensive care to a group of patients (for example based on the patients’ quality of life) would be regarded by some as a form of unfair discrimination.

However, it is not clear that the distinction between treatments and patients withstands scrutiny. This is firstly because a decision to fund a more cost-effective treatment over a less cost-effective treatment necessarily impacts upon groups of patients and conflicts with equality of treatment. So, for example, if the UK NICE decides to fund a novel chemotherapy agent for breast cancer, but not one for prostate cancer, that will necessarily mean that female patients with breast cancer will be able to access a new treatment, while male patients with prostate cancer will not. So the concern about selecting between patients does not yield a distinctive objection against incorporating cost-effectiveness into decision-making about potentially inappropriate treatment.^21^

Secondly, doctors make decisions about subgroups of patients with better or worse prognosis all the time. So, for example, surgery might be offered to patients with locally confined cancer, but not to those with lymph node involvement. A heart transplant might be offered to children with a primary cardiomyopathy, but not to those with multi-organ failure. Doctors already make decisions about providing intensive care to some patients, but not to others on the grounds of being potentially inappropriate and they are already influenced by factors such as the patient’s chance of survival, duration of survival or quality of life. When they make such decisions they are clearly *discriminating*—in the sense that they are choosing. But there is a further question about whether this choice is based on morally relevant characteristics (in which case it would be fair), or morally irrelevant characteristics (in which case this would be unfair discrimination).

One possibility is that including some characteristics to select between patients is more unfair than others. For example, it may be that it would be more justified to withhold treatment on the basis of high cost, or on the basis of low chance of benefit, than on the basis of poor quality of life or short duration of survival. We have discussed elsewhere, how the competing values of fairness and benefit might be balanced in decisions about treatment in intensive care.^22^

One realistic problem for the thresholds derived above in Sects. 4–6 of this paper, is whether they can be applied. It might be accepted, for example, that 6–9% is the right probability threshold for providing 3 weeks of intensive care to an adult patient. However, the challenge will be, for an individual patient, how are we to work out what their chance of survival with treatment actually is? In many cases, it will be extremely difficult to estimate the probability of survival, and there will be a large degree of uncertainty about any figures that are derived.^23^ That uncertainty may be even greater for some types of predictions. For example, predictions of future length of life or quality of life may be very difficult to pin down.

However, this concern does not necessarily negate the value of thresholds such as the ones derived in the preceding parts of this paper. Firstly, in some situations there may be sufficient epidemiological data to calculate estimates of survival. For example, a large US study of extremely premature infants has generated an online prognostic calculator, providing an estimated chance of survival given key variables, (birth weight, sex, gestational age, prior treatment with corticosteroids) (Tyson et al. 2008; Boland et al. 2013).^24^ Secondly, even if prognosis is difficult to precisely estimate, it may be possible to determine that the chance of survival falls within a particular range. As noted above, this could be used to support or to refute claims that treatment of particular patient groups would be potentially inappropriate. Third, while future degrees of impairment may be hard to predict in some cases, in others it will be less uncertain. In the paediatric or adult intensive care setting, evidence of pre-existing impairment is likely to provide a much clearer guide to future quality of life. If it is acceptable to include disability in decisions about resources, it might be more relevant to decisions in the PICU or AICU settings. Finally, while there are likely to be uncertainties, and likely to be particular problems in borderline cases, it seems likely that these concerns apply even more to current use of the concept of potentially inappropriate treatment in intensive care. Even if there are challenges to the use of cost-effectiveness-based thresholds in intensive care, these may nevertheless be better than the status quo.

## Conclusions

In this paper, we have focused on the relevance of resource limits to treatment decisions in intensive care. We have explored the application of cost-effectiveness thresholds to determinations that treatment is potentially inappropriate. We have shown that such thresholds could be used (given certain assumptions) to answer clinically relevant questions about when low probability, or high cost treatment should not be provided, and when quality of life may be too low. We have outlined, but rejected several potential counter-arguments against the use of such thresholds.

The 2015 Consensus statement on potentially inappropriate treatment called for the medical profession to engage in debate with wider society on the appropriate boundaries for medical practice around the end of life (Bosslet et al. 2015). This paper will hopefully contribute to such a debate. The 2015 Consensus statement also noted that such policies would need to provide a high level of detail and specificity to be clinically useful (Bosslet et al. 2015). One strong argument in favour of the explicit cost-effectiveness derived thresholds for treatment developed in this paper, is that it is possible to openly debate them, and, if judged acceptable, apply them clinically in a way that is transparent and consistent.

Although we have derived some specific answers about where the thresholds might lie, these are not designed to be applied directly to clinical practice. They are based on a set of assumptions—about the costs of treatment, about the outcome of therapy, and about the cost-effectiveness threshold, that would need to be assessed and potentially modified in a particular situation. A number of these assumptions may be challenged, and the specific values used may not be correct. We have isolated individual variables to develop thresholds, however, more complex analysis could apply a combination of different clinical and prognostic variables to model the probability of treatment being cost-effective for individual patients or groups of patients. There are other factors that are relevant to clinical decisions, and there may be situations where it would be justified to provide intensive care even where this would appear to be outside cost-effectiveness-based thresholds.^25^ However, our analysis has shown that cost-effectiveness could be used to derive specific answers to previously intangible questions about the limits of appropriate treatment in intensive care. With further analysis, thresholds could be developed that could be applied to clinical decisions, and help make ethically consistent, robust and transparent determinations that treatment is potentially inappropriate.

The foregoing analysis highlights some of the areas where empirical data would be required to inform resource-based decisions about the appropriateness of treatment. It also highlights important questions about disability, quality of life and resource allocation. Cost-effectiveness thresholds rely upon placing a value on survival in a healthy, unimpaired state. The application of cost effectiveness thresholds to intensive care may mean denying life-sustaining treatment to some patients with predicted disability. Furthermore, there remain important unresolved questions about which costs should be included, and whether some treatments are exempt from cost-effectiveness consideration.

Finally, we have examined questions of the appropriateness of treatment in well-resourced countries. We have drawn some comparisons and noted the possible implications of different cost-effectiveness thresholds. However, there is further work to be done on exploring the important questions raised by resource limits for critically ill adults, children and newborn infants in countries with more limited health resources. In such settings there may be a much more significant role for the use of cost-effectiveness-derived thresholds for providing intensive care.

One basic, uncontroversial and logically required principle of ethics is consistency: to treat like cases alike. Cost-effectiveness thresholds are regularly used elsewhere in publicly funded health systems to aid difficult decisions about whether or not life-prolonging medicines should be provided. In the absence of a morally relevant difference, these same thresholds should potentially apply to other life-prolonging treatment. Cost-effectiveness may provide a way to define and determine the appropriateness of intensive, expensive care.

## Abbreviations

AICU: Adult intensive care unit
$\bar{C}$: Mean cost of treatment
$\bar{C}_{\text{d}}$: Mean daily cost of treatment
CET: Cost effectiveness threshold
C_T_: Cost threshold (maximum yearly cost of treatment for treatment to be cost-effective)
$\bar{d}_{s}$: Mean duration of survival (if the patient survives)
D_T_: Duration threshold (minimum duration of survival for treatment to be cost effective)
$\bar{E}$: Mean effectiveness of treatment
GDP: Gross domestic product
GMC: General Medical Council
NICE: National Institute for Health and Care Excellence
NICU: Neonatal intensive care unit
$\bar{p}$: Mean probability of survival
PICU: Paediatric intensive care unit
P_T_: Probability threshold (minimum probability of survival for cost-effective treatment)
$\bar{q}$: Mean health-related quality of life
QALY: Quality adjusted life year
QT: Quality threshold (minimum quality of life consistent with cost-effective treatment)

## Footnotes

1 While our focus is on public healthcare systems that already consider cost-effectiveness for other interventions, there is a strong argument that providing ‘non-costworthy care’ should also usually be avoided in privately funded health insurance schemes. See for example, (Menzel 2003).

2 For example, treating a patient with a highly infectious disease in an open intensive care unit might increase the risk of spread to other sick and immunocompromised patients.

3 In the intensive care unit, one potent cause of moral distress amongst staff is the provision of treatment judged to be futile (Mobley et al. 2007).

4 Countries do not necessarily have a single set threshold. The threshold may be applied within a range of cost-effectiveness. Countries vary in the value of the threshold and in how it is applied. For our purposes, we will assume a conservative threshold of £30,000, however, the analysis below is not dependent on a particular figure. If readers prefer a higher or lower value, that can be substituted in the calculations below. One way of determining cost-effectiveness thresholds for different countries draws on the economic prosperity of a country. The WHO-CHOICE analysis of cost-effectiveness and strategic planning used a value of GDP per capita for therapies judged to be ‘cost effective’, while therapies costing more than three times GDP per capita were non-cost effective (World Health Organisation 2015).

5 The traditional value for the cost-effectiveness threshold in the US has often been quoted as being $50,000 per QALY, though the scientific origin of this figure has been disputed (Grosse 2008).

6 Conversely, if there is a problem with determining whether or not treatment should be provided based on cost effectiveness thresholds, that problem will apply to all treatments—not just in the context of potentially inappropriate treatment in intensive care.

7 A small proportion of patients who have intensive care withdrawn nevertheless survive (Lewis et al. 2007; Brecht and Wilkinson 2015). There will be costs associated with the care of these patients, as well as costs associated with end of life care of patients who have treatment withdrawn. However, the costs and effects of continuing intensive care are highly likely to dominate any analysis, so for the sake of simplicity, we will set those counterfactual possibilities aside.

8 Modelling based economic evaluations often build in a range of different possible outcomes, with probabilities and valuations assigned to each. So, for example, there may be probability p_1_ of surviving for d_1_ years in state of q_1_ quality of life, while there is probability p_2_ of d_2_ years and q_2_ quality of life etc. For this analysis, we will focus on single values for $\bar{p}$, $\bar{d}_{s}$ and $\bar{q}$; this could be conceptualized as representing the average values for a cohort of patients, or the expected probability/duration/quality for a single patient.

9 For simplicity, we will not apply discounting to future costs or the value of future life-gains.

10 These assumptions are clearly generous. We will revisit shortly alternative values for these variables. Here, we are applying estimates of average duration of stay/survival etc. For individual patients, it may be anticipated that these would be longer or shorter than average. Where additional information like this is available, it could be used to assess whether treatment should be provided below the standard probability threshold (or should be declined despite a higher chance of survival). We will return in Sect. 7 to some additional challenges in applying thresholds to individual patients.

11 Median survival from an adult surgical intensive care unit was 10 years (Timmers et al. 2011).

12 This value is on the basis of a cardinal measure of health-related quality of life where 1 represents perfect health and 0 represents death. See Sect. 6 for exploration of reduced quality of life.

13 Cost per day in adult intensive care depends on the number of organs supported. In an adult ECMO trial, modeling used an estimated cost per ICU day (base case) of £1300 (Peek et al. 2010).

14 Again, for simplicity in the examples below we are assuming no net cost, and no benefit if the treatment is not provided (ie that all patients not receiving this treatment will die). For cases where this assumption does not hold, formula 4. compares the additional cost per QALY of the life-saving treatment (compared with the average costs and average health effects of usual care).

15 The corresponding values based on the US threshold (and US costs) would be 333 days for an adult and 2,333 days for a newborn.

16 Expected high mortality in long staying patients potentially leads to withdrawal of treatment, and consequently increased mortality (Wilkinson 2009).

17 One older study suggested that 50% of newborn infants requiring mechanical ventilation for more than 6 months survived to age 3 (Gibson et al. 1988). In a large US cohort study, 50% of extremely low birth weight infants ventilated for more than 4 months survived (Walsh et al. 2005) The probability of survival after prolonged intensive care appears similar in children and adults. One recent study, including a 20 year review of patients staying in paediatric intensive care for more than 1 month found 49.8% survival at follow-up. (Namachivayam et al. 2012) In a medical adult intensive care unit, the likelihood of survival fell from 75% at admission to 50% at day 4. (Meadow et al. 2011) The chance of survival plateaus after this point, however (Kojicic et al. 2011).

18 It is not easy to estimate longevity after prolonged intensive care. One study of prolonged mechanical ventilation (more than 3 weeks) in adult intensive care patients found that 56% were alive after 1 year (Unroe et al. 2010). A study in adult patients receiving prolonged mechanical ventilation ( > 1 week) after cardiac surgery, found a median survival of 6.2 years (Engoren et al. 2000).

19 This is in comparison with the option of withdrawing treatment (with an assumed subsequent quality of life of zero). While it is possible that some patients (treated with mechanical ventilation) may be able to survive without that treatment, and have a quality of life greater than zero—this would potentially reduce the effect gained by treatment, and therefore increase further the incremental cost-effectiveness ratio.

20 There is more that needs to be done to identify whether and why certain types of health-related costs should be excluded from cost-effectiveness analysis. Further analysis of that important question is beyond the scope of this paper.

21 As noted earlier in Sect. 3, a defence of the use cost-effectiveness for deciding between treatments is beyond the scope of this paper.

22 For further exploration of questions of disability discrimination and cost-effectiveness in relation to intensive care see (Wilkinson and Savulescu 2014) and (Wilkinson and Savulescu (forthcoming)).

23 As a simple example, if there have been 100 previous cases of a given condition, and 3 infants have survived, the 95% confidence interval for the proportion of cases surviving ranges from 0 to 6–8% (depending on the statistical model used) (Brown et al. 2001). If there have been 1000 previous cases, the confidence interval ranges from 2–4%. If there have been only 30 previous cases, the upper limit of the confidence interval stretches as high as 18%.

24 While this is only one example, "Big Data" promise to make such estimates more reliable and based on more comprehensive and integrated data sets capable of task specific sub-classification.

25 For example, there may be benefits to other individuals. Clinicians may feel that it would be important to admit a child to intensive care to allow parents to come to terms with the severity of a child’s illness and imminent death, or to allow other family members to attend the patient’s bedside. Alternatively, there may be reason to think that there are non-health benefits to the individual patient that would justify prolonging life (for example allowing them to set their affairs in order or to see a new grandchild). It would be hard, perhaps impossible, for any cost-effectiveness analysis to incorporate benefits of this sort. We acknowledge the need for some flexibility (and compassion) in the application of any thresholds.
